# Hybrid modelling for stroke care: Review and suggestions of new approaches for risk assessment and simulation of scenarios

**DOI:** 10.1016/j.nicl.2021.102694

**Published:** 2021-05-07

**Authors:** Tilda Herrgårdh, Vince I. Madai, John D. Kelleher, Rasmus Magnusson, Mika Gustafsson, Lili Milani, Peter Gennemark, Gunnar Cedersund

**Affiliations:** a Integrative Systems Biology, Department of Biomedical Engineering, Linköping University, 58185 Linköping, Sweden; b Charité Lab for Artificial Intelligence in Medicine – CLAIM, Charité University Medicine Berlin, Germany; c School of Computing and Digital Technology, Faculty of Computing, Engineering and the Built Environment, Birmingham City University, Birmingham, UK; d ADAPT Research Centre, Technological University Dublin, Ireland; e Bioinformatics, Department of Physics, Chemistry and Biology, Linköping University, Sweden; f Estonian Genome Center, Institute of Genomics, University of Tartu, Tartu, Estonia; g Drug Metabolism and Pharmacokinetics, Early Cardiovascular, Renal and Metabolism, BioPharmaceuticals R&D, AstraZeneca, Gothenburg, Sweden

**Keywords:** Stroke, Mechanistic modelling, Machine learning, Bioinformatics, Precision medicine

## Abstract

Stroke is an example of a complex and multi-factorial disease involving multiple organs, timescales, and disease mechanisms. To deal with this complexity, and to realize Precision Medicine of stroke, mathematical models are needed. Such approaches include: 1) machine learning, 2) bioinformatic network models, and 3) mechanistic models. Since these three approaches have complementary strengths and weaknesses, a hybrid modelling approach combining them would be the most beneficial. However, no concrete approach ready to be implemented for a specific disease has been presented to date. In this paper, we both review the strengths and weaknesses of the three approaches, and propose a roadmap for hybrid modelling in the case of stroke care. We focus on two main tasks needed for the clinical setting: a) For stroke risk calculation, we propose a new two-step approach, where non-linear mixed effects models and bioinformatic network models yield biomarkers which are used as input to a machine learning model and b) For simulation of care scenarios, we propose a new four-step approach, which revolves around iterations between simulations of the mechanistic models and imputations of non-modelled or non-measured variables. We illustrate and discuss the different approaches in the context of Precision Medicine for stroke.

## Highlights

• Modelling is needed to deal with the complexity of stroke.
• There exist 3 relevant modelling approaches with complementary strengths and weaknesses: machine learning, large-scale network, and mechanistic models.
• Hybrid modelling can make use of their respective strengths.
• We review these approaches and propose a new hybrid scheme for calculation of stroke risk calculation and simulation of care scenarios.

## 1 Background

### 1.1 The challenge of big data in diseases like stroke warrants the development of new Precision Medicine technologies

**Fig. 1:**
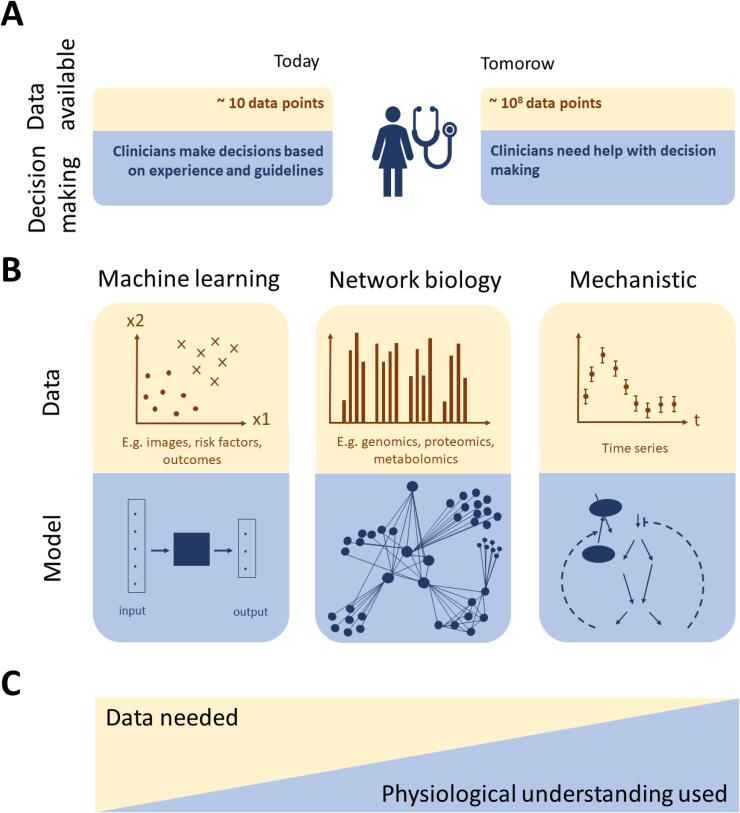
The challenge of big data in clinical practice and clinical data analysis: (A) Today, the physicians manage by conventional inspection and reasoning around data, based on experience and clinical guidelines. Tomorrow, this simple approach will no longer be a feasible solution to properly analyse all data. (B) Overview of the three main types of modelling approaches and the kinds of data they can analyse. (C) Illustration of the amount of data and physiological understanding that are typically used by the different modelling approaches in B. Incorporating physiological understanding, if it exists, in the modelling process can be used to restrict the learning problem and thus limit the amount of data needed. Methods to analyse data for which physiological understanding is lacking, or is not used, usually require larger amounts of data.

The healthcare sector has, like many other parts of society, entered the age of big data. Currently, the standard practice for a physician diagnosing a patient is to look at a handful of biomarkers, interview the patient, and then use his/her experience and clinical guidelines, to decide what to do next (Fig. 1A). Notably, this strategy may soon be augmented by the advent of modern measurement and data storage technologies, since the amount of data available from a patient visit can easily become so large that it cannot be inspected manually, even less analysed. Moreover, we now have access to large clinical studies, with millions of similar patients examined and followed over time, which all serve as important background information. Finally, the patient may also have access to his/her own sensor technologies in a variety of wearables generating huge amounts of data. To ignore the new data and knowledge now available to a physician is not an acceptable solution. Therefore, new technologies, which can make use of and integrate all these data and knowledge types, are urgently needed.

The use of this vast amount of data for highly individualised predictions is called Precision Medicine (PM). PM is a form of healthcare that relies on data, algorithms, and precise molecular tools to offer individualised care for patients. These tools give insight into mechanisms of disease, treatment, and prevention. By treating the patient as an individual, the attending physician can adapt treatment to variations in pathophysiology, genome, and anatomy. PM has the potential to improve outcomes and to reduce healthcare costs. It has for example already been successfully used in oncology, to find genetic mutations, and the approach is now being considered in a variety of different fields (Hinman et al., 2017, Rostanski and Marshall, 2016).

Stroke is a suitable candidate for PM, for all phases of stroke care – prevention, acute treatment, and rehabilitation. Stroke is characterized by a complex pathophysiology, comprising several medical and environmental factors, and involving multiple organs, timescales, and control mechanisms (Rostanski and Marshall, 2016), for which more pathophysiological data relevant for stroke have now become available. Depending on the phase of stroke care, different types of data, like clinical, imaging and psychometric data are available for ischemic stroke, the most common form of stroke. Additionally, given its high prevalence, a lot of data are routinely acquired and can be made available. A PM approach can therefore integrate these data and thereby offer better and personalized care, decision making, and risk calculation (Hinman et al., 2017, Dzau and Ginsburg, 2016).

## 2 Three types of data, and three corresponding types of modelling approaches

The available data relevant for stroke care come in different forms and can be subdivided according to the method by which they can be analysed (Fig. 1B). We here distinguish between three such classes of methods. (I) One set of analysis approaches is known as machine learning (ML), that can generate predictions from mechanistically not understood data without requiring explicit prior domain knowledge (Kelleher et al., 2015). (II) Another set of analysis tools belongs to the fields of omics analysis and bioinformatics. Omics refers to large biological data that, as an example, describe complete genetic (e.g. single nucleotide variants) or molecular (e.g. proteins and mRNA) profiles of an organism. These types of data can be analysed using graph theory, or biological networks, in order to systematically analyse the relation between factors that are found to individually have small effects, e.g. clusters of genes that together show a significantly changed expression pattern (Gustafsson et al., 2014, Kim and Tagkopoulos, 2018, Rappoport and Shamir, 2018). (III) A final example of analytical tools is mechanistic modelling, used to analyse time-resolved data. The underlying biological system is often modelled by ordinary differential equations (ODEs), e.g. models describing glucose- and insulin interplay on the whole-body level (Dalla Man et al., 2007). Another example is blood pressures and flows, which can be described by either zero-dimensional Differential-Algebraic Equations (DAEs), or partial differential equations (PDEs) (Casas et al., 2017, Casas et al., 2018, Frey et al., 2020). Generally, data can also represent intracellular concentrations of metabolites and proteins, which may be described by ODE models that are based on metabolic and signalling pathways (Brännmark et al., 2013, Brännmark et al., 2017, Nyman et al., 2014). The ODE and PDE models can then be simulated to produce data similar to the data produced by the real mechanisms it describes.

Each of the three approaches (Fig. 1B) has its strengths and weaknesses. (I) ML approaches can build models that map between inputs and outputs without knowing anything about the physiological mechanisms in that mapping. Some weaknesses include that the development of ML models relevant for PM, such as artificial neural networks (ANNs), usually require large amounts of training data. Furthermore, these methods do not use or contribute to physiological understanding, and the developed ML models may be biased in regard to what data that was chosen to train the model (i.e. sample bias). (II) The strengths of bioinformatics approaches are that they can develop quasi-phenomenological models in cases where too few samples have been collected to use more data-heavy ML approaches. This is done by combining the raw data with prior knowledge describing for instance tentative networks of interactions. However, bioinformatics approaches normally aim to develop coarse-grained models that may contain several false positive predictions, and such approaches are thus not to be considered as fully mechanistic. (III) The mechanisms incorporated in mechanistic models mathematically describe the underlying biochemical interactions and how the experimentally observed dynamics could have been produced. Systematic hypothesis testing and model refinement ultimately result in a high-quality system understanding, but this highly time-consuming approach is only possible in cases where prior domain knowledge and the right type of data are available. Note that these large-scale bioinformatic networks differ from other networks models, such as deep ANNs, in that nodes in bioinformatic network models represent actual physical connections between physical entities, for instance genes or proteins, while the nodes in the middle layers of ANNs usually do not represent physiological entities in that sense.

### 2.1 The need for hybrid modelling

The above comparison between ML models, bioinformatics models, and mechanistic models quickly reveals that they have complementary strengths and weaknesses. In other words, different types of data require different types of analysis methods. The strongest possible models would therefore arguably be hybrid models, since they can combine the strengths of the different modelling approaches, and can make use of all types of data.

Some hybrid model approaches have been proposed for biomedical applications. One of those proposals came from the Discipulus network, which produced a roadmap for how “Digital Patients” - *in silico* representations of individuals - can be developed (Díaz et al., 2020, Viceconti et al., 2016). This roadmap outlines many useful ideas ranging from data integration and handling, to modelling approaches, and even to clinical applications. However, the network did not develop any concrete models, even though some existing non-hybrid models for stroke are mentioned in the roadmap. There are also some specific hybrid models developed for biological applications, summarized in e.g. these reviews (Doyle et al., 2013, Stéphanou et al., 2016). From these reviews, at least three conclusions can be drawn: i) hybrid models are still rare, but their incidence has been rapidly increasing over the last couple of years (Stéphanou et al., 2016); ii) there is no consensus regarding nomenclature, but some basic options for how the models can be combined (Fig. 6A) are emerging; iii) there are no fully developed hybrid models that combine mechanistic multi-level models with ML and bioinformatics in a clinically useful way, and especially none for stroke.

### 2.2 Purpose and outline of review

**Fig. 2:**
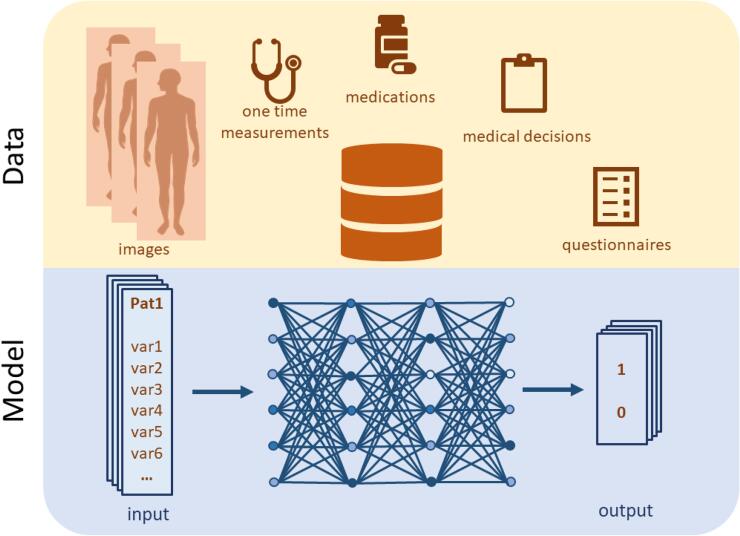
ML approaches exemplified by supervised learning in the form of an ANN. The network is trained to predict outputs from a set of input data by changing the weights of the network depending on differences between model output and data. Input data typically consists of patient characteristics, such as images and medications. Output data often consists of outcome, e.g. whether the patient has suffered a stroke or not within a specific time period. If the ANN is used for imputation, the output data instead consists of a patient variable.

**Fig. 3:**
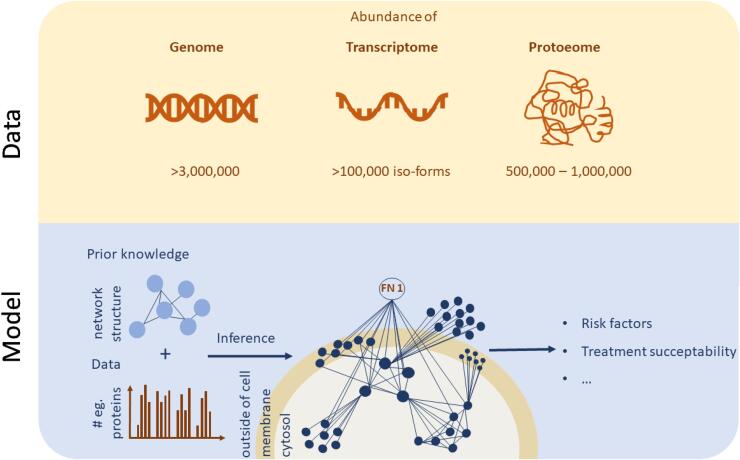
Biomarker discovery from high throughput biological data using bioinformatic network models. These networks to some extent describe real biological interaction networks between the involved molecules in a disease aetiology. In the figure, such a network is exemplified as a network describing protein interactions and their abundance inside, in the membrane of, and outside cells. As can be seen, the resulting network has an internal structure: it contains hubs and clusters whose degree of abundance can be associated with a certain disease or treatment susceptibility.

**Fig. 4:**
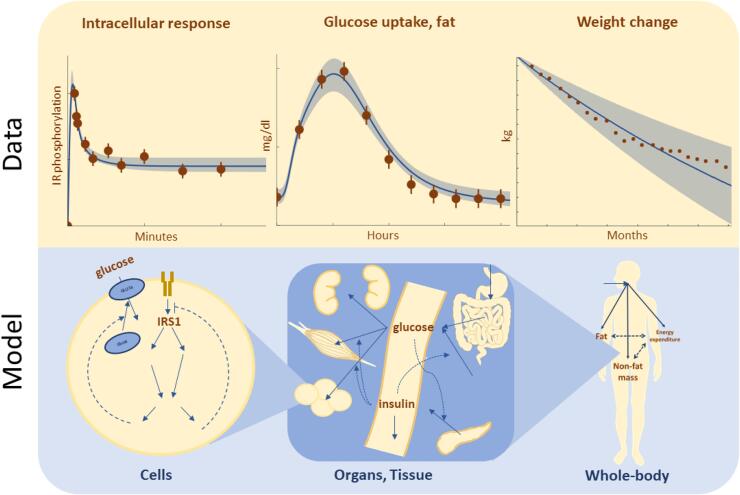
Overview of the components and structure of multi-time scale, multi-level, and mechanistic models. Here, we exemplify the structure of these kinds of models by an interconnected model describing whole-body weight regulation (Hall et al., 2011), glucose and insulin homeostasis (Dalla Man et al., 2007), and intracellular insulin signalling in fat cells (Brännmark et al., 2013, Nyman et al., 2011). This combined model can simulate what happens on a time-scale of minutes, hours, and months, and with an uncertainty (light blue) dependent on its fit to data. (For interpretation of the references to colour in this figure legend, the reader is referred to the web version of this article.)

**Fig. 5:**
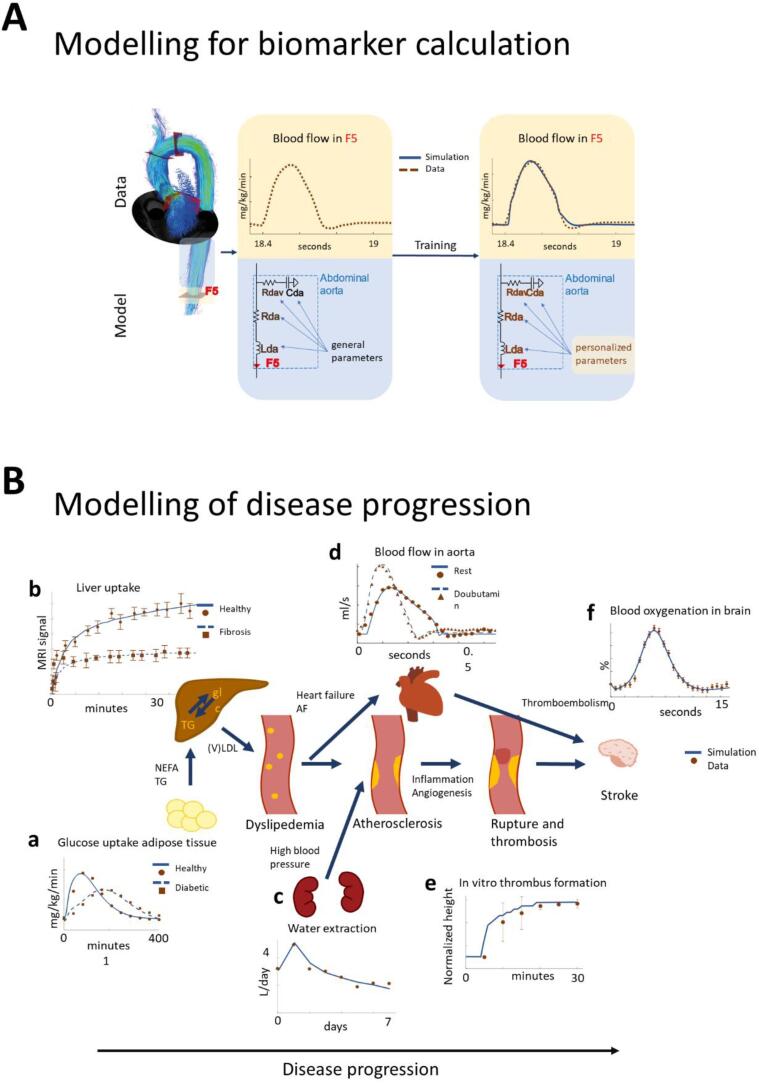
Abilities of mechanistic models. (A) The basic principle by which biomarkers can be calculated from data using mechanistic models, here illustrated by modelling of vessels around the aorta using a standard Windkessel model and 4D flow MRI data (Casas et al., 2017, Casas et al., 2018). Here, only a part of the model is shown, the one describing blood flow in the abdominal aorta. The general parameters are taken from literature. The personalized parameters received by training of the entire model can then be interpreted as personalized values of biomarkers. In the illustration, these biomarkers are the resistance (Rda), inertance (Lda), and capacitance (Cda) of the abdominal aorta, and the viscoelastic resistance (Rdav) of Cda. All of these are mechanistic and interpretable biomarkers, not available in the raw data, but whose values can be estimated using the mechanistic model. (B) Overview of some of the most important sub-models that could form the basis of a stroke simulation model, with agreements between simulations (-,--) and data (dots) in most of the main processes leading up to a stroke. (a) A model for adipose tissue meal response (at t = 0) in healthy controls (-) and type 2 diabetes patients (--)^12^; (b) Liver models describing liver uptake of a contrast agent injected at t = 0, in patients ranging from healthy controls-) to advanced fibrosis--)^94^; (c) The kidney water extraction response following administration of an SGLT2 inhibition drug at t = 0^95^; (d) Blood flow in aorta for normal healthy subjects (-) and for subjects perturbed with Dobutamine (--)^10^; (e) Thrombus formation in an in vitro vessel initiated at t = 0^96^; (f) The Blood Oxygen Level Dependent signal in response to brain activity (Sten et al., 2017). These models are currently isolated from each other, but could become a part of an interconnected stroke simulation model. Such interconnected simulations should still be different from patient to patient.

**Fig. 6:**
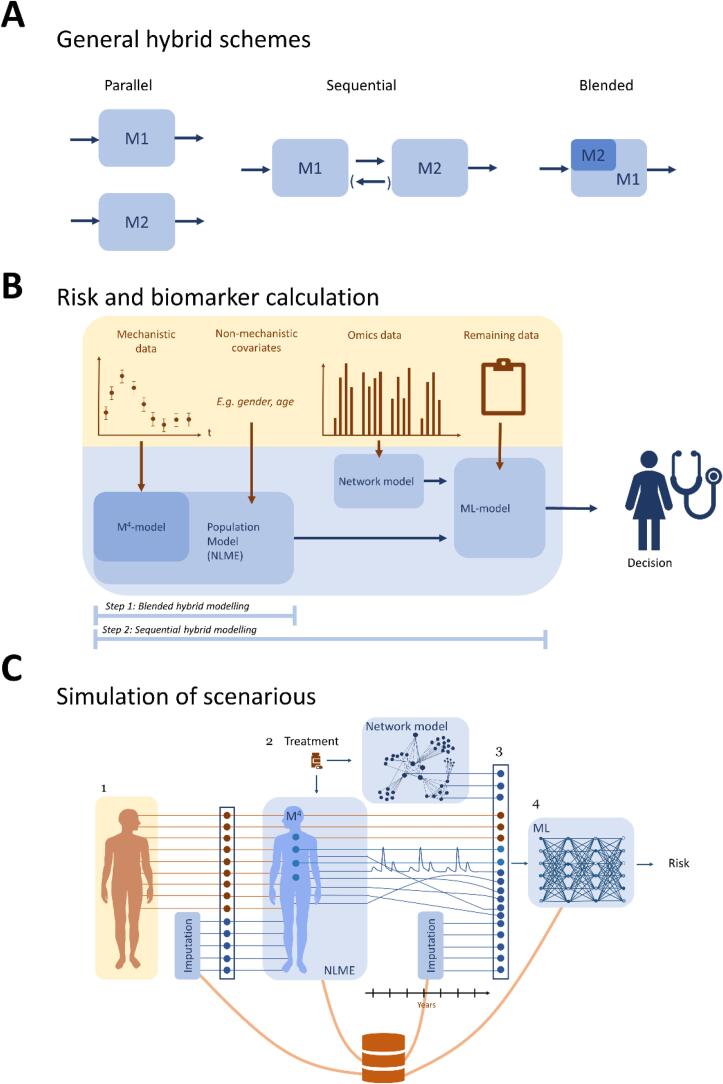
Hybrid modelling (A) The three main approaches to hybrid modelling most often adopted today. Note that the denomination of these approaches has not yet converged, and that other coexisting names for these and related approaches have been proposed (Stéphanou et al., 2016). For instance, sequential modelling is sometimes referred to as iterative or staged hybrid modelling, depending on if the feedback from M2 to M1 is included. (B) Schematic outline of our approach for hybrid modelling, when calculating risk scores of the patient at the time of examination. (C) Outline of our approach to simulation of scenarios, using our new hybrid models.

There is thus a strong need to develop the field of hybrid modelling. Herein, we present a roadmap for how hybrid models could be developed. Whereas it could be noted that the methodologies and strategies are general, and therefore could be applied to other diseases as well, we primarily illustrate their usage for stroke care. Specifically, we propose a hybrid methodology which can be used to a) calculate risk factors, and b) simulate different disease scenarios (e.g. the progression based on different interventions, or no intervention). The next section provides a slightly more in-depth review of the three different modelling approaches (Fig. 2, Fig. 3, Fig. 4), a closer look at their different strengths and weaknesses, and some state-of-the-art models relevant for stroke (Fig. 4, Fig. 5). We also propose a specific combination of some models for this purpose. Thereafter, we propose a two-step approach for the calculation of risk scores (Fig. 6B), and a four-step approach for simulation of scenarios (Fig. 6C). Finally, we discuss the implications of the proposed hybrid approach and outline some of the remaining challenges.

## 3 Review: Existing modelling methodologies and concepts

### 3.1 Machine learning and heterogenous, large-scale data

ML is the subfield of Artificial Intelligence focused on developing algorithms that induce models from data without requiring explicit prior domain knowledge (Kelleher et al., 2015, Lip et al., 2010). The two main classes of ML are supervised and unsupervised learning. In the case of supervised learning, pairs of inputs and outputs are known, and the learned model provides a mapping of inputs to outputs (Fig. 2). In the case of unsupervised learning, input data is not predefined, and instead, the underlying structure of the data is searched for by the algorithm (Alpaydin, 2009). The in- and outputs of ML models are usually labelled as either discrete or continuous. Discrete labels, or classes, can for example be whether a patient has a certain disease or not, and the prediction of whether a patient belongs to such a class is done by a classification model. In contrast, continuous labels (such as abundance of a specific mRNA^21^), can be predicted by regression models.

The field incorporates many kinds of approaches, with different needs regarding the amount of data amount and the required physiological understanding. For the purpose of using hybrid modelling for PM, approaches that do benefit a lot of data are usually preferred, e.g. since there exists a lot of relevant and heterogenous data (individual and populational) lacking physiological understanding that can be used to constrict the learning. For calculating of risk scores, supervised learning can be used.

There are numerous supervised learning methods available in biomedicine, with different amounts of data needed (Breiman, 2001, Margolin et al., 2006). Here, we give a short explanation on two popular supervised learning methods for predictive model generation in biomedicine, on separate sides of the amount of data needed: support vector machines (SVMs) and ANNs (Warwick, 2013).

Both SVMs and ANNs map between known inputs and outputs (Alpaydin, 2009). For model training, a set of data with known classification is given to the algorithms. The final model assigns new data into one of the different classes. ANNs require larger datasets to do this mapping than SVMs. The basic idea of SVMs is to identify a discriminating line that separates entities belonging to different classes (Kelleher et al., 2015). ANNs are structured as interconnected networks of nodes (Wasserman, 1993). When training to data, ANNs update weights describing the strength of interaction between the nodes, to describe input–output relationship in the best possible way (Warwick, 2013). ANNs have recently gained in popularity through the specific subtype known as deep ANNs, also called deep learning models, which are ANNs with two or more intermediary layers of nodes (Kelleher, 2019). Such deep ANNs have for instance been able to classify images on the same level as a highly trained radiologist (Becker et al., 2017), and are recurrently proposed to advance the field of PM (Grapov et al., 2018, Ching et al., 2018).

## 4 Strengths and weaknesses

A key benefit of ML approaches is that they normally do not require understanding of the processes involved, i.e. they do not need any prior domain knowledge; they are therefore sometimes referred to as phenomenological models. Phenomenological models primarily learn patterns between the phenomena recorded in the training data. In other words, these methods are, by themselves, purely data-driven, and can generate e.g. a network or statistical mapping from inputs to outputs from the data directly. The fact that they are data-driven means that they can find patterns not yet found by humans, and they can also make use of data that is not yet mechanistically understood.

One of the drawbacks of these approaches is that they often require, or at least hugely benefit from, large number of datapoints, to be able to extract information without using *a priori* knowledge. Dealing with large amounts of data is not only complicated due to difficulties in amassing such data, but also due to difficulties in properly understanding such large amounts of data. Importantly, the user must understand the data enough to be able to properly trust it. This data quality check can to some extent be done by saving some data for cross-validation: if the data can be correctly predicted, that argues for the existence of useful information and a sufficient quality in the data. Another critical parameter characterizing the size of the data is the number of features, i.e. patient variables. As measurement technologies evolve, we can measure an increasing number of patient variables in routine examinations and clinical studies. This ever-increasing number of features limits our ability to extract relevant personalized information from big data, even using ML methodologies (Zhou et al., 2017). One main reason behind this limitation is that ML models require more training samples than the number of parameters to be learned, further increasing the amount of data needed. Another reason is that more features make it more difficult to incorporate multiple patients into one disease class, since they also get more dissimilar with increased number of features (Barbour, 2019). This fact that the number of features often scales badly with the complexity of the model is known as the curse of dimensionality (Bellman, 1984).

Another drawback with ML models is the need to set hyperparameters – parameters that cannot be learned from data, and instead must be set before the learning process begins. These include for example the number of layers and neurons of a deep ANN, or the kernel parameters in SVMs. To identify the optimal hyperparameters for a specific learning task, a systematic search for the optimal hyperparameters is performed, often using a so-called grid search. Doing a grid search can be computationally expensive, particularly using deep ANNs (Kelleher, 2019). There is at present no strong principles to inform these decisions, and instead, one must rely on heuristic rules.

One last drawback with ML models are that they on their own do not make use of or add to the available physiological knowledge about the system, i.e. they lack explainability. Giving physiological reasons for predictions and decisions is highly important in the clinical context of stroke, where motivation is crucial for creating trust for both patient and medical practitioners. Explainability can also aid in improving generalisation by using domain knowledge to constrain the learning problem. While there are several ongoing developments to help improve the interpretability of ML approaches, i.e. ability to show how a prediction was reached (Medicine TLR, 2018, Voosen, 2017), there are still few examples available for explainability in stroke (Zihni et al., 2019). Finally, another way of improving both interpretability and explainability of ML approaches is the hybrid modelling approach, proposed herein, by combining ML models with more explainable model types, such as mechanistic models.

Given these strengths and weaknesses, we want to highlight two tasks where ML can be relevant for stroke care, and especially in a hybrid modelling context: 1) imputation of missing data, and 2) risk calculation. We will now explain in more detail how ML can be applied for these two tasks.

### 4.1 Useful ML abilities in hybrid modelling of stroke: Imputation and risk calculation

Missing data are commonplace in clinical data sets (Wood, 2004, Burnett et al., 2011), and is especially problematic when applying data-driven methods such as ML (Livne et al., 2017, Livne et al., 2018, Obermeyer and Emanuel, 2016, Zitnik et al., 2019). One reason for this challenge is the fact that ML approaches are, as discussed above, dependent on the quality of data. Usually, missing data are handled by simply discarding features or patients with missing data. However, discarding patients can lead to biased results, and further diminishing the size of clinical datasets which may already be too small (Liu and Gopalakrishnan, 2017, Demissie et al., 2003). Similarly, discarding features might lead to missing important risk factors. Therefore, a more robust method for handling missing data is needed. Such methods are centred on imputation of the missing data.

Imputation of missing data can be done using various ML approaches, by estimating non-measured variables with likely values. The simplest, but still commonly used, imputation methods use population average values, or sampling (hot-deck imputation). More advanced methods commonly used on clinical datasets include expectation maximization (King et al., 2001) and multiple imputation by chained equations (Azur et al., 2011). ML approaches for imputation could also develop specific models (such as Hopfield Networks, or Energy Based Models) for how to map the measured variables to the non-measured variables, by considering the measured variables as inputs, and the non-measured variables as outputs. Which imputation method should be used ultimately depends on the dataset, and different methods will influence the performance of data-driven predictive models differently (Kossen et al., 2019).

Another aspect of ML methods that is useful for hybrid modelling of stroke is the ability to calculate risk assessment. Risk assessments are done routinely in the clinic, mainly to assess and choose an appropriate treatment or preventative measure. In stroke care, it could be the risk of having a stroke, the risk of a bad outcome after acute stroke, the chance of improving function by following a certain treatment, etc. Both discrete and continuous supervised ML can be used to produce such risk assessments. These risks can also have an accompanying uncertainty, depending on e.g. the quality of data. Finally, the predictive quality of the model is assessed, for example by using a Receiver Operating Characteristic (ROC) curve, which measures how well the model balances false positives and false negatives.

### 4.2 Examples of ML for stroke

ML is, in the context of stroke care, most frequently used for image analysis. For example, ML has been used to predict the location of the final infarct on a voxel-by-voxel basis, which is important for predicting the outcome of the stroke (Livne et al., 2018, Nielsen et al., 2018). It is also within image analysis that the only commercial applications of ML for stroke treatment are found: For example, Viz.ai© detects large vessel occlusions by using deep ANNs, and Infervision© estimates volume, stroke type, and stroke location using an ML-based decision support system.

For predicting the risk of having a stroke or another cardiovascular event in the future, there are no commercial examples, but there are several reported in the literature, and a few already used in the clinic. For assessing the risk of stroke or other cardiovascular diseases, clinics currently simple engines based on e.g. linear regression are used, such as the Framingham risk score (D’Agostino et al., 2008, Seshadri et al., 2010) and SCORE (Conroy et al., 2003). More advanced models that have been proposed include different kinds of ANNs (Quesada et al., 2019, Zarkogianni et al., 2018, Huang et al., 2018, Shanmugam et al., 2019), the Bayesian network type and Naïve Bayes (Quesada et al., 2019, Li et al., 2016), decision trees (Li et al., 2016, Hung et al., 2017), and random survival forests (Ambale-Venkatesh et al., 2017, Jamthikar et al., 2019, Al-Mallah et al., 2017). Some have predicted stroke for specific subgroups, either because of a group’s increased risk, or because of difficulties of detecting the risk in those groups (Zarkogianni et al., 2018, Li et al., 2016). Some of the above studies are based on fairly large prospective cohorts, of 5000 participants and up (Shanmugam et al., 2019, Ambale-Venkatesh et al., 2017, Al-Mallah et al., 2017). Features used for these models include the same ones used routinely in clinic, such as high blood pressure, smoking, diabetes, age, and history of stroke (Quesada et al., 2019). Other studies also use more unconventional features, such as ECG signals (Shanmugam et al., 2019), claims data (Hung et al., 2017), and other types of heterogenous data (Zarkogianni et al., 2018, Huang et al., 2018, Li et al., 2016, Ambale-Venkatesh et al., 2017, Jamthikar et al., 2019), and some assess how many features to optimally include in different ways (Huang et al., 2018, Ambale-Venkatesh et al., 2017). Although some sort of combination of different ML models (e.g. ensemble strategies) exists (Zarkogianni et al., 2018, Shanmugam et al., 2019), hybrid modelling approaches using other types of models are still uncommon. For a more comprehensive review of ML for stroke care, see (Mouridsen et al., 2020).

### 4.3 Bioinformatic network models and omics

The field of bioinformatics aims to analyse and interpret high-throughput biological datasets – omics datasets – using mathematical and statistical methods (Yang et al., 2017). Common types of omics datasets include (Tseng et al., 2015): complete genomes - genomics; proteomes - proteomics; gene expression - transcriptomics; and - small molecule metabolites - metabolomics (Sidorov et al., 2019). Such omics measurements provide a snapshot of the physiology of a sample from a human body, and can be analysed using ML approaches. However, more often, such omics datasets are used to infer models representing the underlying structure of biological interactions, in the forms of networks.

The basic data structure of a network is a graph. The nodes in the graph represents biochemical species (e.g. genes or proteins), and the connections between them, usually referred to as edges, represent the interactions between the nodes (Barabási and Oltvai, 2004). Often, these graphs are either constructed using curated databases, reverse-engineered from omics data, or combinations thereof. There are multiple curated databases, such as TRRUST for gene regulation (Han et al., 2018), PhosphoSitePlus for phosphoproteomic regulation (Hornbeck et al., 2014), and STRING for protein–protein interaction networks (Szklarczyk et al., 2019). However, the information in these databases may be based on weakly defined associations, such as two variables often co-occurring in the same texts, obtained from text mining (Harmston et al., 2010). In contrast, network inference from data has the ability to capture biological features that are unique to a certain data sample (Zou and Hastie, 2005). Note that these large-scale bioinformatic networks differ from other networks models, such as deep ANNs, in that nodes in bioinformatic models represent actual physical connections between e.g. genes, while the nodes in ANNs usually do not represent physiological entities in that sense, and require a lot of data to be powerful.

These networks have been shown to have a skewness among their interactors to a few numbers of genes, so-called hubs. Similarly, the networks typically show co-localisation of interactors to certain areas corresponding to gene network modules. These facts have been used by translational bioinformatics researchers to focus the identification to hubs and modules and test their relevance to diseases (Gustafsson et al., 2014, Barabási and Oltvai, 2004). The derived modules and genes contain many genes which then can be validated by genomic concordance. Such genomic concordances are said to be found if the module derived from gene expression and protein interactions are enriched with disease-associated single nucleotide polymorphisms (SNPs) from Genome-Wide Association Studies (GWAS). Apart from enrichment-based validations, candidate groups of genes are supported by clinical and functional studies. These validation and network approaches were applied e.g. in (Gustafsson et al., 2014, Magnusson and Gustafsson, 2019).

## 5 Strengths and weaknesses

The main advantages of the biological network approach are its direct use of prior domain knowledge, and that the automatic inference from large data sets results in a useful network overview with direct biological interpretation (Fig. 3). Another advantage with the network and bioinformatics approach is that the identification of hubs and clusters may serve as more robust and sensitive biomarkers, compared to e.g. individual genes. For instance, it may be that all genes in a hub are changed too little to be significant when considered individually, but where the clustering of the genes in such a hub allows for detection of the changes anyway, since sufficiently many of them change in a way that is consistent with model predictions of such changes. In the same way, non-consistent changes in a cluster may remove biologically meaningless noise in individual genes.

Current network methodologies also have important shortcomings. For example, most network models contain numerous false positives, are biased towards some well-studied genes, and are estimated to miss as much as 80% of all true interactions (Menche et al., 2015). A last and important shortcoming is the steady-state approximation of an adaptive system, which can be a major concern for systems with mixed slow and fast temporal processes. One alternative that solves this problem is the development of mechanistic models, as discussed in Section 2.3.

Bioinformatic network models can be developed based on personal data, and can as such be used to assess an individual’s predisposition of having a stroke and his/her susceptibility to a treatment. We will now describe the application of bioinformatic network models for stroke care further.

### 5.1 Useful abilities of network models in hybrid modelling of stroke: New biomarkers and risk scores

A person’s predisposition to stroke is rarely caused by one gene, but rather by a combinations of genes (Markus, 2010, Barabási et al., 2011, van der Wijst et al., 2018), with varying risks depending on the specific stroke subtype (Floßmann et al., 2004). It is also believed that there are different genetic predispositions for stroke and many subtypes of stroke with different genetic makeups (van der Wijst et al., 2018, Markus, 2012). Which genetic variation is underlying a stroke influences drug metabolism, and so affects drug efficacy and potential side-effects (Feero et al., 2011). Furthermore, genetic variations causing inter-individual variation in drug responses are also affected by environmental exposure, and affected by which cell types are involved in the subtype (van der Wijst et al., 2018). Personalizing bioinformatic network models can therefore be essential in assessing an individualized stroke risk.

Using patient-specific omics data, a patient-specific genetic, transcriptomic, proteomic or metabolomic network can be developed. The personalised network can be compared with healthy and diseased ones, to identify risk of having a stroke or adverse outcomes of a stroke. For treatment susceptibility, a personal network could also be describing the cellular dysfunction underlying the stroke, to see if a drug or combination of drugs actually induce detectable change in the network activity (Barabási et al., 2011, van der Wijst et al., 2018). Furthermore, network modelling can be used to find driver genes, findings that can be used for targeted stroke treatment (van der Wijst et al., 2018).

### 5.2 Examples of bioinformatic network models for stroke

Using omics data together with network modelling has led to improved prognostic stratification in stroke (Goldenberg et al., 2014), and discovery of several important metabolite-stroke associations (Sidorov et al., 2019). For example, a study by Muñoz et al. used a clinical proteomics approach to identify candidate biomarkers, for both stroke diagnosis and stroke rehabilitation (Muñoz et al., 2018). This demonstrated a fundamental role of fibrinogen plasmatic levels on patient admission to the stroke rehabilitative care. Specifically, these levels correlated with a gain in activities of daily living at discharge from rehabilitative care (Muñoz et al., 2018).

Genetic risk prediction has been used for targeted disease prevention in subgroups of individuals at extremely high risk for disease before any symptoms manifest. For example, GWAS have been very successful in identifying novel genetic variants associated with various common complex diseases (Visscher et al., 2017). The MEGAstroke consortium has collected 29 cohorts with a total of 67,162 individuals with ischaemic stroke and 454,450 controls. By *meta*-analysing the results of the GWAS, they added 22 new stroke risk loci to the previously known 10 genes (Malik et al., 2018). This was further updated with the discovery of three more risk loci in 2019 (Malik et al., 2018), bringing the total number of genes associated with stroke to 35. Creating genomic risk scoresGRS) based on the associated variants has only shown modest results for stroke compared to established risk factors (Rutten-Jacobs et al., 2018). However, a more advanced *meta*-scoring approach, incorporating GWAS summary statistics for stroke and its aetiological subtypes together with GWAS summary statistics for risk factors and co-morbidities of ischemic stroke proved more successful (Abraham et al., 2019). The metaGRS was validated in UK biobank where the individuals in the top 0.25% of the metaGRS had a three-fold higher risk of stroke. Genetic risk prediction has also been used in a personalised manner. For example, a study by Wong et al. analysed gene expression in the blood of post-stroke patients to distinguish the cause of stroke, and then used the resulting networks to predict stroke aetiology and outcome (Wong et al., 2015).

### 5.3 Mechanistic models and dynamic data

A mechanistic model is essentially a computational implementation of a mechanistic hypothesis, and thus enables that hypothesis to be quantitatively tested (Kitano, 2002). Mechanistic models thereby also use domain theory to constrain the learning problem, to decrease computational power, and to increase generalizability and interpretability of the model. The entire model structure describes a specific mechanistic hypothesis of how a system functions. The unknown parameter values in the model are then estimated by fitting the model to available estimation data, within physiologically realistic ranges. If the model cannot produce an acceptable agreement with the estimation data, it and the underlying mechanistic hypotheses are rejected. This question is formally tested using statistical tests, such as a chi-squared test (Cedersund and Roll, 2009). A model that can agree with estimation data is then analysed to find predictions with uncertainties. Well-determined predictions, sometimes called core-predictions (Cedersund, 2012), are interesting because they describe features that must be fulfilled in the model, and they can also be used to design new experiments, and to test the model by comparing the predictions with new validation data. In this way, mechanistic modelling can aid experimental hypothesis testing: to draw conclusions from existing data and design new experiments.

Another usage of mechanistic modelling is to combine validated models for sub-systems, e.g. organs, with each other in larger models. The resulting interconnected model with crosstalk between many organs can be simulated on both short and long timescales. An example of what such a model can do is seen in Fig. 4. On the long-term timescale (months), the model can describe slow processes like weight-loss. On the shorter timescale (hours), the model can describe how a meal is digested, by e.g. looking at the time-varying glucose uptake in one of the organs. On the shortest timescale (minutes), one can look at how intracellular processes, such as protein signalling phosphorylation events, happen within seconds or minutes after a hormone has reached a target cell.

As of now, most mechanistic models are trained and validated on population data, but the interconnected model can also be trained and validated on individual data, thus personalising it. Example of such personalised models exist, e.g. models for prediction of variations in glucose meal responses (Kovatchev et al., 2009). It is also possible to connect or translate the behaviours in such an interconnected model with a corresponding model for mice, by establishing a scaling between them. Scaling between mice and human models enables knowledge gained from experiments done on living mice models to translate properly to human functions (Alskär et al., 2017). For brevity, such mechanistic, multi-level, multi-timescale, and multi-species can be called M^4^ models.

## 6 Strengths and weaknesses

The benefits and weaknesses of mechanistic models are almost exactly the inverse of that of ML and bioinformatic network models. Some of the most important benefits are that it is possible to input physiological knowledge; that one can work with small informative datasets; and that model predictions can always be traced back to the exact generating mechanism in the system. Another important benefit is that mechanistic models can be reused, and easily expanded in new contexts, as new data and applications become available.

Some of the most important weaknesses of mechanistic modelling are that they themselves are usually not statistical in nature, i.e. they usually do not naturally produce such things as risk scores. Similarly, uncertainty in structural prior information is seldom formally represented in mechanistic models. Furthermore, most systems biology mechanistic models are so-called mean value models, and advanced patient-specific modelling requires the expansion of purely mechanistic modelling to also include phenomenological covariates, as is done in nonlinear mixed-effects modelling (Jonsson et al., 2000, Karlsson et al., 2015). The latter method is one of the hybrid modelling approaches discussed further below.

Just as for ML and network models, mechanistic models have useful abilities in a hybrid modelling of stroke: 1) predicting the time-evolution of key biomarkers in a patient-specific manner, and 2) predicting the time-evolutions of these biomarkers for different scenarios, e.g. different treatments.

Useful abilities of mechanistic models in hybrid modelling of stroke: personalised predictions of new mechanistic biomarkers and simulations of scenarios*.*

The basic principle by which new biomarkers can be obtained from the combination of a mechanistic model and mechanistically understood data is depicted in Fig. 5A. The example is taken from (Casas et al., 2017), but the principle is general. The data in this example is sampled by a 4D flow Magnetic Resonance Imaging (MRI) technique. From this sampled data, the flows through each point in space can be determined at all time-points. These point-based estimates can be aggregated, and the various black planes in Fig. 5 indicate areas in the vessel system where a quantification of the flow is made. Each such quantification leads to a time-series of the flows. The magnitude of the spatially dependent flow is depicted in the top left graph in Fig. 5A, and the quantified time-series through the plane F5 is depicted in brown time-series in the middle of Fig. 5A. Once data, such as these, have been acquired, they are then used to estimate the parameters in the mechanistic model.

The mechanistic model in this example is a zero-dimensional ODE model that describes how the blood flow propagates from each compartment in the model to the next, based on various physical properties of the vessels, such as volume, stiffness, and compliance. This type of model is based on an analogy between blood flows and electrical circuits, and so-called Windkessel models (Fig. 5A). The model is fitted to individual time-series data to generate patient-specific parameters, and a personalized model. In the fitting process, a cost function quantifies the distance between the experimental data (Fig. 5A, brown dashed line) and the simulations (blue solid line), for each tested parameter combination, and a standard numerical optimization algorithm uses this cost function to estimate the optimal model parameters (Cedersund et al., 2016). The uncertainty of the obtained patient-specific parameter values can be obtained using e.g. MCMC sampling or profile likelihood methods (Cedersund, 2012, Cedersund et al., 2016).

Values that are obtained using mechanistic models are not values inherently available in the raw data: the prior information of physical processes encoded by the mechanistic model and the inference machinery are required, in combination with the raw data, to obtain these values. In this example, the predicted physical properties of the vessels (compliance, stiffness, volumes, etc) are new biomarkers, which provide additional information about the patient. They are useful when one e.g. seeks to calculate the risk that the patient will suffer a stroke: the stiffer the vessels, and the smaller the effective volumes, the higher risk for the patient to suffer a cardiovascular event.

Finally, mechanistic models that have been personalized can be used to simulate different scenarios, such as a new treatment or a prevention measure, to see their predicted effect. This way of simulating different scenarios is useful for designing new interventions and/or understanding why an intervention is performing poorly on a certain patient.

### 6.1 Examples of mechanistic models relevant for stroke

For stroke prevention, there is no complete model that simulates all the different processes involved in the ethiology leading up to a stroke. Nevertheless, most of the underlying processes have been modelled (Fig 5B). These processes include e.g. dysfunctional glucose homeostasis, diabetes, and complications of dyslipidemia such as non-alcoholic fatty liver disease and liver fibrosis (Fig 5B, a-b) (Brännmark et al., 2013, Conroy et al., 2003, Forsgren et al., 2019); impacts on the vasculature, including blood-pressure and volume regulations, e.g. in response to diabetes drugs inhibiting Sodium-Glucose Transport 2SGLT2 (Fig 5B, c-d) (Casas et al., 2018, Hallow et al., 2018); and the final thrombosis (Fig 5B, e) (Taylor et al., 2016) which leads to thromboembolisms in the vasculature of the brain (Fig 5B, f), described by models of the neurovascular coupling (Sten et al., 2017) or network/multi-level models describing the electrophysiology of the brain (Newton et al., 2018, Hagen et al., 2016, Nair et al., 2014). The stroke itself can me modelled as spreading depression, where near complete depolarization of neurons throughout the brain, as in (Newton et al., 2018). Another relevant modelling endeavour is that of aging, which effects all stages of progression towards a stroke (Borgqvist et al., 2020). These models could be interconnected into a single simulation model, which also simulates the cross-talk between all of these processes.

For stroke treatment, there are models for prediction of clinical outcome. Livne et. al has developed a biophysiological model of brain perfusion that integrates individual patient-specific imaging data and boundary conditions (e.g. blood pressure, intracranial pressure) (Livne et al., 2017). This model computes flow volumes, velocities, and perfusion pressures for different brain areas. This is the first brain circulation model that integrates individual stroke patient neuroimaging data and enables simulation of brain blood flow and perfusion based on vascular pathology such as stenosis and occlusion. The model also allows incorporating vessel segments into the model to examine their effect in the cerebral vasculature and/or to supplement missing medical data/information. Importantly, this model is based on routine clinical imaging. Thus, this technology can provide individual data for a PM approach, without the necessity of additional time-consuming and even potentially harmful approaches used today in perfusion imaging.

### 6.2 General schemes for hybrid modelling

We have now seen three different modelling approaches – machine learning, bioinformatic network models and mechanistic models – each with their own weaknesses and strengths. Let us end this review part, by looking at some of the main schemes available for combinations of different types of models, i.e. hybrid modelling (Fig. 6A). The first option is parallel models, where there is no cross-talk between the constituent models. The second option is called sequential hybrid modelling, which is done if the output of one of the two models serves as input to the other model. This approach is sometimes also called a staged hybrid model. Sequential modelling can also be done in a reciprocal manner, where the output to the second model serves as the input to the first model, for each step in the model simulations. Such models are also called iterative hybrid models. Finally, the last option for hybrid modelling is called blended modelling, in which the two models have been fully merged with each other, into a combined model.

### 6.3 Proposed hybrid modelling approaches

Using the general hybrid modelling schemes, we will now describe how we propose to combine ML, bioinformatic network models, and multi-level mechanistic modelling for stroke care. We mainly use preventative care as a possible example, but we believe that the algorithm could be used for acute and rehabilitative stroke care as well. These hybrid approaches constitute a roadmap for hybrid modelling within stroke care. We propose two different approaches to obtain two of the main tasks needed for clinical usage: a) calculation of risk factors, and b) simulation of scenarios.

### 6.4 Prediction of risk factors and outcomes

Our proposed hybrid modelling approach for calculating risk scores and outcomes is outlined in Fig. 6B. As can be seen, we propose to use two types of hybrid modelling schemes: blended and sequential (compare with Fig. 6A).

In Step 1, blended hybrid modelling is achieved by introducing a statistical and phenomenological component into mechanistic models. Specifically, we propose using a modelling methodology called Nonlinear Mixed-Effects Modelling (NLME). With NLME models, there is the possibility to introduce covariates into otherwise normal nonlinear mechanistic ODE models. These covariates impact the value of the parameters in a phenomenological fashion, as illustrated by the following two examples:

Say that some parameters in the mechanistic blood flow model (Fig. 5A) are dependent on gender. The data used for parameter estimation would then be subdivided into two parts: one for males and one for females. Each gender would get a specific distribution of the parameter where the covariate is specified as having an effect.

Age impacts the mechanistic parameters (e.g. the compliance), but say that we do not yet understand all the mechanisms involved in this dependency. In that case, the age should be introduced as a covariate. How the covariate is impacting the parameter is then postulated as a phenomenological formula (e.g. saturated dependency if it reaches a maximum, or a step-wise if there is a sudden jump in an affect at a specific age).

NLME models describe the distribution of each parameter across the population. This distribution is normally described by a covariance matrix, which specifies the width of the distribution and how each parameter is correlated with the other parameters. In practice, this covariance matrix is obtained at the same time as the other parameters for each individual patient, by formulating a joint likelihood function in the estimation step, and by introducing the additional assumption that the parameter values across the population should follow a certain predefined type (e.g normal or log-normal distribution). This additional assumption implies that the parameters will be more well-determined in the case of non-informative data (where the data for an individual patient is insufficient to have well-determined values for all parameters), as demonstrated in e.g. (Karlsson et al., 2015).

In Step 2, Sequential hybrid modelling, all the new biomarkers are combined in an ultimate risk score. These biomarkers could have been obtained using either NLME combined with the mechanistic models (Step 1), and/or using the bioinformatic network modelling (Fig. 3). Apart from these biomarkers, there are all the biomarkers and data that have not yet been used, or that still may have useful information (Fig. 6B). All these data and biomarkers are then combined into a classical ML model, such as an ANN (see Section 2.1), which calculates a risk. As an example, for preventative care, the ML algorithm can be trained on large prospective clinical studies where both all or many of the biomarkers are measured, and where patient outcome is available. The main outcome of interest is then whether the patient will suffer a stroke within the next pre-defined time-period.

### 6.5 Simulation of scenarios

The second type of usage for our hybrid modelling we propose is for simulation of individual patient scenarios. Our strategy for how these simulations will be done is outlined in Fig. 6C. As can be seen, there are four major steps involved.

Step 1: *Set up a personalized* M^4^ *model.* All available data for the patient is identified. The rest of the information that can be used to further personalize the M^4^ model are imputed by a ML model based on large cross-sectional data. For instance, for one patient with known age and gender, 4D flow MRI data and some metabolic plasma levels are available, while blood pressure and fatty acid levels are not, and these are thus imputed. In Fig. 6C, the available data are represented by red dots, and the imputed variables as blue dots. The measured and imputed data are then fed to the NLME and M^4^-model. To simulate the M^4^-model, it also needs parameters that are specific to the model and that usually are not, or even cannot, be measured directly, such as different mass flow velocities or levels of insulin sensitivity. In Fig. 6C, these values are symbolized by dots in blue inside the M^4^-model. Some such parameters can be trained from the available data (as in Fig. 5A). Other parameters can be taken from literature. These approaches imply an uncertainty in the chosen values, meaning that many sets of parameters will be available for the same patient. Note that the resulting M^4^-model is personalized.

Step 2: *Simulation of scenarios.* The personalized M^4^-model is used to investigate different scenarios. Such scenarios could be introducing different medications or alterations in diet. These simulations are read out, and the relevant biomarkers and other interesting model properties are analysed. Predicted biomarkers can go into the outcome calculations, but there are many other variables that could be interesting and useful to investigate. For example, if the model predicts a decrease in inflammation and body fat upon a change in diet, one would then like to know why these positive effects, which will lower the risk of having a stroke, are seen. That question can be answered by inspecting more detailed simulations, which reveal e.g. the precise mechanisms and processes involved in responding to the diet, and how these mechanisms come into play for this patient.

Step 3: *Set up ML model for risk calculation.* Data for a timepoint in the future is fed to the ML risk calculation model. This data includes values from relevant biomarkers simulated by the M^4^-model in step 2, new imputed values, static or otherwise known measured values (such as age), and corresponding information from bioinformatic network models.

Step 4: *Risk calculation.* A risk for a specific outcome is calculated, for each interesting timepoint in the simulated scenario. This could be the last timepoint, or several intermediate ones as well. These risk calculations are done in the same as in Fig. 6B. After this, Steps 2, 3, and 4 could be repeated to identify the corresponding risk for scenarios, which allows us to infer for instance how the risk of suffering a stroke changes given different simulated scenarios. Given models for acute treatment or rehabilitation of stroke, one can in the same way see how other statistical properties, such as the likelihood of improving a particular function, is expected to change upon different treatments.

Using these four steps, one obtains a personalized model for a patient, also in the case of non-complete data; the model can in such cases be used to simulate various scenarios, and to physiologically answer why the obtained results are predicted; and the model can anyway calculate the updated risk at each timepoint in the simulated scenarios. In this usage, all different types of data and knowledge are combined. This combination of data and knowledge is only possible with the usage of a hybrid modelling scheme, such as the ones outlined here.

There are other functions that could be incorporated into a hybrid modelling scheme that were not discussed here. For example, ML models could be used to choose between different mechanistic models. Some biological systems act according to different mechanisms in different individuals, sometimes depending on if they have a disease, and sometimes arbitrarily, and thus there can exist several models for the same system. ML methods could then be used to either find the best model for a particular patient from existing ones, or maybe even to develop models semi-automatically as in (King et al., 2004, King et al., 2009). ML can also be used for feature selection, as in e.g. (Li et al., 2016), delimiting the features used for the risk calculation, and they can be used for extracting relevant information from images.

## 7 Summary

In this review, we have outlined how ML, bioinformatic networks, and mechanistic models can be combined to be helpful in stroke care. The background section outlines why hybrid modelling is such a beneficial idea. In Section 2, we reviewed existing modelling approaches: ML (Fig. 2), bioinformatic networks ML (Fig. 3), multi-level mechanistic models (Fig. 4, Fig. 5), and available hybrid combinations (Fig. 6A). In Section 3, we outlined how combining these three modelling approaches can be done to predict outcome (Fig. 6B). The main idea is to do initial analysis of mechanistically understood data and covariates using NLME approaches (which constitutes a blended hybrid modelling approach), to analyse omics data using bioinformatic network models (also leading to predictions of new biomarkers), and then combine all of the new and old biomarkers in a ML model, which gives the ultimate risk calculation. Finally, in Fig. 6C, we outlined a four-step approach to how one can use hybrid modelling to simulate scenarios: to answer the question of *why,* from a physiological stand-point, the predicted scenarios are believed to be the outcome, and to estimate the risk at each timepoint in such simulated scenarios. For the case of stroke care, this outlined approach could for example be used to calculate someone’s risk for having a stroke, given nonmodifiable risk factors (e.g. age, sex), other conditions related to stroke risk (e.g. diabetes, hypertension), and current life-style (e.g. smoking, diet). Depending on how high the estimated risk is, the approach can then be used to evaluate what intervention (e.g. anticoagulant or treating hypertension) would be most effective, given that persons predispositions, genetic or other, by comparing what intervention decreases the risk the most. The physiological reasoning behind the estimated risks made by the model can be scrutinized by looking at the simulated scenarios, what biomarkers have evolved and how (e.g. blood pressure or plasma glucose levels).

With these new type of hybrid models, it will be possible to develop digital twins, i.e. personalized models. These digital twins can be useful in healthcare for a wide variety of uses: to aid doctor-patient communication and pedagogics, to help motivate patients to follow prescribed treatments, to inspire people to do more preventive or restorative measures, and to help the doctor devise a personalized diagnosis or treatment plan. In this manuscript, we have outlined a concrete methodological basis for such digital twin developments. Looking forward in time, it is clear that while the parts necessary for hybrid modelling already exists, as in the different models and modelling techniques applied in the way we suggest here, their combination and validation on data still must be done. New clinical studies to validate the crosstalk between the different aspects of the models are ongoing, and will be done during the next couple of years. After that, the digital-twin models need to be embedded in eHealth apps and products, which also need to be validated for clinical usage. In practice, this means that a digital twin for stroke care may be between 5 and 10 years away.

## CRediT authorship contribution statement

**Tilda Herrgårdh:** Writing - original draft, Visualization. **Vince I. Madai:** Writing - original draft, Writing - review & editing. **John D. Kelleher:** Writing - original draft, Writing - review & editing. **Rasmus Magnusson:** Writing - review & editing. **Mika Gustafsson:** Writing - review & editing. **Lili Milani:** Writing - review & editing. **Peter Gennemark:** Writing - original draft, Writing - review & editing. **Gunnar Cedersund:** Writing - original draft, Writing - review & editing.

## References

[b0005] Abraham G., Malik R., Yonova-Doing E., Salim A., Wang T., Danesh J. (2019). Genomic risk score offers predictive performance comparable to clinical risk factors for ischaemic stroke. Nat. Commun..

[b0010] Al-Mallah MH, Elshawi R, Ahmed AM, Qureshi WT, Brawner CA, Blaha MJ et al. Using Machine Learning to Define the Association between Cardiorespiratory Fitness and All-Cause Mortality (from the Henry Ford Exercise Testing Project). Am J Cardiol 2017; 120: 2078–2084. 10.1016/j.amjcard.2017.08.029 28951020

[b0015] Alpaydin E. (2009). Introduction to machine learning. MIT press.

[b0020] Alskär O., Karlsson M.O., Kjellsson M.C. (2017). Model-based interspecies scaling of glucose homeostasis: Model-based interspecies scaling of glucose homeostasis. CPT Pharmacomet. Syst. Pharmacol..

[b0025] Ambale-Venkatesh B, Yang X, Wu CO, Liu K, Hundley WG, McClelland R et al. Cardiovascular Event Prediction by Machine Learning: The Multi-Ethnic Study of Atherosclerosis. Circ Res 2017; 121: 1092–1101. 10.1161/CIRCRESAHA.117.311312 PMC5640485 28794054

[b0030] Azur M.J., Stuart E.A., Frangakis C., Leaf P.J. (2011). Multiple imputation by chained equations: what is it and how does it work?: Multiple imputation by chained equations. Int. J. Methods Psychiatr. Res..

[b0035] Barabási A-L., Oltvai Z.N. (2004). Network biology: understanding the cell’s functional organization. Nat. Rev. Genet..

[b0040] Barabási A-L., Gulbahce N., Loscalzo J. (2011). Network medicine: A network-based approach to human disease. Nat. Rev. Genet..

[b0045] Barbour D.L. (2019). Precision medicine and the cursed dimensions. Npj Digit. Med..

[b0050] Becker A.S., Marcon M., Ghafoor S., Wurnig M.C., Frauenfelder T., Boss A. (2017). Deep learning in mammography: diagnostic accuracy of a multipurpose image analysis software in the detection of breast cancer. Invest. Radiol..

[b0055] Bellman R. (1984). Dynamic programming.

[b0060] Borgqvist J., Welkenhuysen N., Cvijovic M. (2020). Synergistic effects of repair, resilience and retention of damage determine the conditions for replicative ageing. Sci. Rep..

[b0065] Brännmark C., Nyman E., Fagerholm S., Bergenholm L., Ekstrand E.-M., Cedersund G. (2013). Insulin signaling in type 2 diabetes. J. Biol. Chem..

[b0070] Brännmark C., Lövfors W., Komai A.M., Axelsson T., El Hachmane M.F., Musovic S. (2017). Mathematical modeling of white adipocyte exocytosis predicts adiponectin secretion and quantifies the rates of vesicle exo- and endocytosis. J Biol Chem.

[b0075] Breiman L. (2001). Random forests. Mach. Learn..

[b0080] Burnett S.J., Deelchand V., Franklin B.D., Moorthy K., Vincent C. (2011). Missing Clinical Information in NHS hospital outpatient clinics: prevalence, causes and effects on patient care. BMC Health Serv Res.

[b0085] Casas B., Lantz J., Viola F., Cedersund G., Bolger A.F., Carlhäll C.-J. (2017). Bridging the gap between measurements and modelling: a cardiovascular functional avatar. Sci. Rep..

[b0090] Casas B., Viola F., Cedersund G., Bolger A.F., Karlsson M., Carlhäll C.-J. (2018). Non-invasive assessment of systolic and diastolic cardiac function during rest and stress conditions using an integrated image-modeling approach. Front. Physiol..

[b0095] Cedersund G. (2012). Conclusions via unique predictions obtained despite unidentifiability - new definitions and a general method: Conclusions via unique predictions obtained despite unidentifiability. FEBS J..

[b0100] Cedersund G., Geris L., Gomez-Cabrero D. (2016). Prediction Uncertainty Estimation Despite Unidentifiability: An Overview of Recent Developments. Uncertainty in Biology.

[b0105] Cedersund G., Roll J. (2009). Systems biology: model based evaluation and comparison of potential explanations for given biological data: Model based evaluation in systems biology. FEBS J..

[b0110] Cedersund G., Samuelsson O., Ball G., Tegnér J., Gomez-Cabrero D., Geris L., Gomez-Cabrero D. (2016). Optimization in Biology Parameter Estimation and the Associated Optimization Problem. Uncertainty in Biology.

[b0115] Ching T, Himmelstein DS, Beaulieu-Jones BK, Kalinin AA, Do BT, Way GP et al. Opportunities and obstacles for deep learning in biology and medicine. J R Soc Interface 2018; 15: 20170387. 10.1098/rsif.2017.0387 PMC5938574 29618526

[b0120] Conroy R.M., Pyörälä K., Fitzgerald A.P., Sans S., Menotti A., De Backer G. (2003). Estimation of ten-year risk of fatal cardiovascular disease in Europe: The SCORE project. Eur Heart J.

[b0125] D’Agostino RB, Vasan RS, Pencina MJ, Wolf PA, Cobain M, Massaro JM et al. General cardiovascular risk profile for use in primary care: the Framingham Heart Study. Circulation 2008; 117: 743–753. 10.1161/CIRCULATIONAHA.107.699579 18212285

[b0130] Dalla Man C., Rizza R.A., Cobelli C. (2007). Meal simulation model of the glucose-insulin system. IEEE Trans. Biomed. Eng..

[b0135] Demissie S., LaValley M.P., Horton N.J., Glynn R.J., Cupples L.A. (2003). Bias due to missing exposure data using complete-case analysis in the proportional hazards regression model. Stat. Med..

[b0140] V. Díaz M. Díaz V, Viceconti M, Stroetmann V, Kalra D. Digital Patient Roadmap. DISCIPULUS Proj Horiz 2020https://www.vph-institute.org/discipulus.html.

[b0145] Doyle O.M., Tsaneva-Atansaova K., Harte J., Tiffin P.A., Tino P., Diaz-Zuccarini V. (2013). Bridging paradigms: hybrid mechanistic-discriminative predictive models. IEEE Trans. Biomed. Eng..

[b0150] Dzau V.J., Ginsburg G.S. (2016). Realizing the full potential of precision medicine in health and health care. JAMA.

[b0155] Feero W.G., Guttmacher A.E., Wang L., McLeod H.L., Weinshilboum R.M. (2011). Genomics and drug response. N. Engl. J. Med..

[b0160] Floßmann E., Schulz U.G.R., Rothwell P.M. (2004). Systematic review of methods and results of studies of the genetic epidemiology of ischemic stroke. Stroke.

[b0165] Forsgren MF, Karlsson M, Dahlqvist Leinhard O, Dahlström N, Norén B, Romu T et al. Model-inferred mechanisms of liver function from magnetic resonance imaging data: Validation and variation across a clinically relevant cohort. PLOS Comput Biol 2019; 15: e1007157. 10.1371/journal.pcbi.1007157 PMC6613709 31237870

[b0170] Frey D., Livne M., Leppin H., Akay E.M., Aydin O.U., Behland J. (2020). A precision medicine framework for personalized simulation of hemodynamics in cerebrovascular disease. Neurology.

[b0175] Goldenberg N.A., Everett A.D., Graham D., Bernard T.J., Nowak-Göttl U. (2014). Proteomic and other mass spectrometry based “omics” biomarker discovery and validation in pediatric venous thromboembolism and arterial ischemic stroke: Current state, unmet needs, and future directions. PROTEOMICS – Clin Appl.

[b0180] Grapov D., Fahrmann J., Wanichthanarak K., Khoomrung S. (2018). Rise of deep learning for genomic, proteomic, and metabolomic data integration in precision medicine. OMICS J. Integr. Biol..

[b0185] Gustafsson M., Nestor C.E., Zhang H., Barabási A.-L., Baranzini S., Brunak S. (2014). Modules, networks and systems medicine for understanding disease and aiding diagnosis. Genome Med.

[b0190] Hagen E, Dahmen D, Stavrinou ML, Lindén H, Tetzlaff T, van Albada SJ et al. Hybrid Scheme for Modeling Local Field Potentials from Point-Neuron Networks. Cereb Cortex 2016; 26: 4461–4496. 10.1093/cercor/bhw237 PMC6193674 27797828

[b0195] Hall KD, Sacks G, Chandramohan D, Chow CC, Wang YC, Gortmaker SL et al. Quantification of the effect of energy imbalance on bodyweight. The Lancet. 2011. doi:10.1016/S0140-6736(11)60812-X. 10.1016/S0140-6736(11)60812-X PMC3880593 21872751

[b0200] Hallow K.M., Helmlinger G., Greasley P.J., McMurray J.J.V., Boulton D.W. (2018). Why do SGLT2 inhibitors reduce heart failure hospitalization? A differential volume regulation hypothesis. Diabetes Obes. Metab..

[b0205] Han H, Cho J-W, Lee S, Yun A, Kim H, Bae D et al. TRRUST v2: an expanded reference database of human and mouse transcriptional regulatory interactions. Nucleic Acids Res 2018; 46: D380–D386. 10.1093/nar/gkx1013 PMC5753191 29087512

[b0210] Harmston N., Filsell W., Stumpf M.P.H (2010). What the papers say: Text mining for genomics and systems biology. Hum. Genomics.

[b0215] Hinman J.D., Rost N.S., Leung T.W., Montaner J., Muir K.W., Brown S., Arenillas J.F., Feldmann E., Liebeskind D.S. (2017). Principles of precision medicine in stroke. J. Neurol. Neurosurg. Psychiatry.

[b0220] Hornbeck PV, Zhang B, Murray B, Kornhauser JM, Latham V, Skrzypek E. PhosphoSitePlus, 2014: mutations, PTMs and recalibrations. Nucleic Acids Res 2015; 43: D512–D520. 10.1093/nar/gku1267 PMC4383998 25514926

[b0225] Huang Z., Dong W., Duan H., Liu J. (2018). A regularized deep learning approach for clinical risk prediction of acute coronary syndrome using electronic health records. IEEE Trans. Biomed. Eng..

[b0230] Hung C.-Y., Chen W.-C., Lai P.-T., Lin C.-H., Lee C.-C. (2017). Comparing deep neural network and other machine learning algorithms for stroke prediction in a large-scale population-based electronic medical claims database.

[b0235] Jamthikar A, Gupta D, Khanna NN, Saba L, Araki T, Viskovic K et al. A low-cost machine learning-based cardiovascular/stroke risk assessment system: integration of conventional factors with image phenotypes. Cardiovasc Diagn Ther 2019; 9: 420–430. 10.21037/cdt.2019.09.03 PMC6837917 31737514

[b0240] Jonsson E.N., Karlsson M.O., Wade J.R. (2000). Nonlinearity detection: Advantages of nonlinear mixed-effects modeling. AAPS Pharm. Sci..

[b0245] Karlsson M., Janzén D.L.I., Durrieu L., Colman-Lerner A., Kjellsson M.C., Cedersund G. (2015). Nonlinear mixed-effects modelling for single cell estimation: When, why, and how to use it. BMC Syst. Biol..

[b0250] Kelleher J.D. (2019). Deep learning.

[b0255] Kelleher J.D., Mac Namee B., D’arcy A. (2015). Fundamentals of machine learning for predictive data analytics: algorithms, worked examples, and case studies. MIT press.

[b0260] Kim M., Tagkopoulos I. (2018). Data integration and predictive modeling methods for multi-omics datasets. Mol. Omics.

[b0265] King RD, Whelan KE, Jones FM, Reiser PGK, Bryant CH, Muggleton SH et al. Functional genomic hypothesis generation and experimentation by a robot scientist. Nature 2004; 427: 247–252. 10.1038/nature02236 14724639

[b0270] King RD, Rowland J, Oliver SG, Young M, Aubrey W, Byrne E et al. The Automation of Science. Science 2009; 324: 85–89. 10.1126/science.1165620 19342587

[b0275] King G., Honaker J., Joseph A., Scheve K. (2001). Analyzing incomplete political science data: An alternative algorithm for multiple imputation. Am. Polit. Sci. Rev..

[b0280] Kitano H. (2002). Computational systems biology. Nature.

[b0285] Kossen T., Livne M., Madai V.I., Galinovic I., Frey D., Fiebach J.B. (2019). A framework for testing different imputation methods for tabular datasets. Neuroscience.

[b0290] Kovatchev B.P., Breton M., Dalla Man C., Cobelli Cl. (2009). *In Silico* preclinical trials: A proof of concept in closed-loop control of type 1 diabetes. J. Diabetes Sci. Technol..

[b0295] Li X., Liu H., Du X., Zhang P., Hu G., Xie G. (2016). Integrated machine learning approaches for predicting ischemic stroke and thromboembolism in atrial fibrillation. AMIA Annu. Symp. Proc. AMIA Symp..

[b0300] Lip G.Y.H., Nieuwlaat R., Pisters R., Lane D.A., Crijns H.J.G.M. (2010). Refining clinical risk stratification for predicting stroke and thromboembolism in atrial fibrillation using a novel risk factor-based approach. Chest.

[b0305] Liu Y., Gopalakrishnan V. (2017). An overview and evaluation of recent machine learning imputation methods using cardiac imaging data. Data.

[b0310] Livne M., Kossen T., Madai V.I., Zaro-Weber O., Moeller-Hartmann W., Mouridsen K. (2017). Multiparametric model for penumbral flow prediction in acute stroke. Stroke.

[b0315] Livne M., Boldsen J.K., Mikkelsen I.K., Fiebach J.B., Sobesky J., Mouridsen K. (2018). Boosted tree model reforms multimodal magnetic resonance imaging infarct prediction in acute stroke. Stroke.

[b0320] Magnusson R., Gustafsson M. (2019). LiPLike: Towards gene regulatory network predictions of high-certainty. Bioinformatics.

[b0325] Malik R., Chauhan G., Traylor M., Sargurupremraj M., Okada Y., Mishra A. (2018). Multiancestry genome-wide association study of 520,000 subjects identifies 32 loci associated with stroke and stroke subtypes. Nat. Genet..

[b0330] Malik R, Rannikmäe K, Traylor M, Georgakis MK, Sargurupremraj M, Markus HS et al. Genome-wide meta-analysis identifies 3 novel loci associated with stroke. Ann Neurol 2018; 84: 934–939. 10.1002/ana.25369 PMC6644297 30383316

[b0335] Margolin A.A., Nemenman I., Basso K., Wiggins C., Stolovitzky G., Favera R.D. (2006). ARACNE: An algorithm for the reconstruction of gene regulatory networks in a mammalian cellular context. BMC Bioinf..

[b0340] Markus H.S. (2010). Unravelling the genetics of ischaemic stroke. PLoS Med.

[b0345] Markus H.S. (2012). Stroke genetics: prospects for personalized medicine. BMC Med.

[b0350] Menche J, Sharma A, Kitsak M, Ghiassian SD, Vidal M, Loscalzo J et al. Uncovering disease-disease relationships through the incomplete interactome. Science 2015; 347: 1257601–1257601. 10.1126/science.1257601 PMC4435741 25700523

[b0355] Mouridsen K, Thurner P, Zaharchuk G. Artificial Intelligence Applications in Stroke. Stroke 2020; 51: 2573–2579. 10.1161/STROKEAHA.119.027479 32693750

[b0360] Muñoz R, Santamaría E, Rubio I, Ausín K, Ostolaza A, Labarga A et al. Mass Spectrometry-Based Proteomic Profiling of Thrombotic Material Obtained by Endovascular Thrombectomy in Patients with Ischemic Stroke. Int J Mol Sci 2018; 19: 498. 10.3390/ijms19020498 PMC5855720 29414888

[b0365] Nair A.G., Gutierrez-Arenas O., Eriksson O., Jauhiainen A., Blackwell K.T., Kotaleski J.H. (2014). Modeling intracellular signaling underlying striatal function in health and disease. *Prog. Mol. Biol. Transl. Sci*. Elsevier.

[b0370] Newton A.J.H., McDougal R.A., Hines M.L., Lytton W.W. (2018). Using NEURON for reaction-diffusion modeling of extracellular dynamics. Front. Neuroinform..

[b0375] Nielsen A., Hansen M.B., Tietze A., Mouridsen K. (2018). Prediction of tissue outcome and assessment of treatment effect in acute ischemic stroke using deep learning. Stroke.

[b0380] Nyman E., Brännmark C., Palmér R., Brugård J., Nyström F.H., Strålfors P. (2011). A hierarchical whole-body modeling approach elucidates the link between in vitro insulin signaling and in vivo glucose homeostasis. J. Biol. Chem..

[b0385] Nyman E., Rajan M.R., Fagerholm S., Brännmark C., Cedersund G., Strålfors P. (2014). A single mechanism can explain network-wide insulin resistance in adipocytes from obese patients with type 2 diabetes. J Biol Chem.

[b0390] Obermeyer Z., Emanuel E.J. (2016). Predicting the future — Big data, machine learning, and clinical medicine. N Engl. J. Med..

[b0395] Quesada JA, Lopez‐Pineda A, Gil‐Guillén VF, Durazo‐Arvizu R, Orozco‐Beltrán D, López-Domenech A et al. Machine learning to predict cardiovascular risk. Int J Clin Pract 2019; 73. doi:10.1111/ijcp.13389. 10.1111/ijcp.13389 31264310

[b0400] Rappoport N., Shamir R. (2018). Multi-omic and multi-view clustering algorithms: review and cancer benchmark. bioRxiv.

[b0405] Rostanski S.K., Marshall R.S. (2016). Precision medicine for ischemic stroke. JAMA Neurol..

[b0410] Rutten-Jacobs LC, Larsson SC, Malik R, Rannikmäe K, MEGASTROKE consortium, International Stroke Genetics Consortium et al. Genetic risk, incident stroke, and the benefits of adhering to a healthy lifestyle: cohort study of 306 473 UK Biobank participants. BMJ 2018; 363: k4168. 10.1136/bmj.k4168 PMC6199557 30355576

[b0415] Seshadri S, Beiser A, Pikula A, Himali JJ, Kelly-Hayes M, Debette S et al. Parental Occurrence of Stroke and Risk of Stroke in Their Children: The Framingham Study. Circulation 2010; 121: 1304–1312. 10.1161/CIRCULATIONAHA.109.854240 PMC2860311 20212282

[b0420] Shanmugam D, Blalock D, Guttag J. Multiple Instance Learning for ECG Risk Stratification. ArXiv181200475 Cs Stat 2019.http://arxiv.org/abs/1812.00475 (accessed 10 Dec2019).

[b0425] Sidorov E., Sanghera D.K., Vanamala J.K.P. (2019). Biomarker for ischemic stroke using metabolome: A clinician perspective. J. Stroke.

[b0430] Sten S., Lundengård K., Witt S.T., Cedersund G., Elinder F., Engström M. (2017). Neural inhibition can explain negative BOLD responses: A mechanistic modelling and fMRI study. NeuroImage.

[b0435] Stéphanou A., Volpert V., Volpert V. (2016). Hybrid modelling in biology: A classification review. Math. Model Nat. Phenom..

[b0440] Szklarczyk D, Gable AL, Lyon D, Junge A, Wyder S, Huerta-Cepas J et al. STRING v11: protein–protein association networks with increased coverage, supporting functional discovery in genome-wide experimental datasets. Nucleic Acids Res 2019; 47: D607–D613. 10.1093/nar/gky1131 PMC6323986 30476243

[b0445] Taylor J.O., Meyer R.S., Deutsch S., Manning K.B. (2016). Development of a computational model for macroscopic predictions of device-induced thrombosis. Biomech. Model Mechanobiol..

[b0450] The Lancet Respiratory Medicine (2018). Opening the black box of machine learning. Lancet Respir. Med..

[b0455] Tseng G., Ghosh D., Zhou X.J. (2015). Integrating omics data.

[b0460] van der Wijst M.G.P., de Vries D.H., Brugge H., Westra H.-J., Franke L. (2018). An integrative approach for building personalized gene regulatory networks for precision medicine. Genome Med.

[b0465] Marco Viceconti, James Kennedy, Adriano Henney, Markus Reiterer, Sebastian Polak, Dirk Colaert et al. in silico Clinical Trials: How Computer Simulation will Transform the Biomedical Industry. .

[b0470] Visscher P.M., Wray N.R., Zhang Q., Sklar P., McCarthy M.I., Brown M.A. (2017). 10 years of GWAS discovery: biology, function, and translation. Am. J. Hum. Genet..

[b0475] VoosenJul. 6 P, 2017, Pm 2:00. How AI detectives are cracking open the black box of deep learning. Sci. AAAS. 2017.https://www.sciencemag.org/news/2017/07/how-ai-detectives-are-cracking-open-black-box-deep-learning (accessed 27 Feb2019).

[b0480] Warwick K. (2013). Artificial intelligence: the basics.

[b0485] Wasserman P.D. (1993). Advanced methods in neural computing.

[b0490] Wong Y-H, Wu C-C, Lai H-Y, Jheng B-R, Weng H-Y, Chang T-H et al. Identification of network-based biomarkers of cardioembolic stroke using a systems biology approach with time series data. BMC Syst Biol 2015; 9: S4. 10.1186/1752-0509-9-S6-S4 PMC4674888 26679092

[b0495] Wood A.M (2004). Comparison of imputation and modelling methods in the analysis of a physical activity trial with missing outcomes. Int. J. Epidemiol..

[b0500] Yang A., Troup M., Ho J.W.K. (2017). Scalability and validation of big data bioinformatics software. Comput. Struct. Biotechnol. J..

[b0505] Zarkogianni K., Athanasiou M., Thanopoulou A.C., Nikita K.S. (2018). Comparison of machine learning approaches toward assessing the risk of developing cardiovascular disease as a long-term diabetes complication. IEEE J. Biomed. Health Inform..

[b0510] Zhou L., Pan S., Wang J., Vasilakos A.V. (2017). Machine learning on big data: Opportunities and challenges. Neurocomputing.

[b0515] Zihni E., Madai V.I., Livne M., Galinovic I., Khalil A.A., Fiebach J.B. (2019). Opening the black box of artificial intelligence for clinical decision support: A study predicting stroke outcome. Health Informatics.

[b0520] Zitnik M, Nguyen F, Wang B, Leskovec J, Goldenberg A, Hoffman MM. Machine learning for integrating data in biology and medicine: Principles, practice, and opportunities. Inf Fusion 2019; 50: 71–91. 10.1016/j.inffus.2018.09.012 PMC6242341 30467459

[b0525] Zou H., Hastie T. (2005). Regularization and variable selection via the elastic net. J. R. Stat. Soc. Ser. B Stat. Methodol..

